# MYH knockdown in pancreatic cancer cells creates an exploitable DNA repair vulnerability

**DOI:** 10.1016/j.neo.2025.101138

**Published:** 2025-02-11

**Authors:** James Ephraums, Janet Youkhana, Aparna S. Raina, Grace Schulstad, Kento Croft, Amanda Mawson, John Kokkinos, Estrella Gonzales-Aloy, Rosa Mistica C. Ignacio, Joshua A. McCarroll, Cyrille Boyer, David Goldstein, Marina Pajic, Koroush S. Haghighi, Amber Johns, Anthony J. Gill, Mert Erkan, Australian Pancreatic Cancer Genome Initiative (APGI), Phoebe A. Phillips, George Sharbeen

**Affiliations:** a Pancreatic Cancer Translational Research Group, School of Biomedical Sciences, Lowy Cancer Research Centre, UNSW Sydney; NSW 2052, Australia; b Australian Centre for Nanomedicine (ACN), UNSW Sydney, Australia; c Children's Cancer Institute, Lowy Cancer Research Centre, UNSW Sydney; NSW 2052, Australia; d Garvan Institute of Medical Research; NSW 2010, Australia; e Cluster for Advanced Macromolecular Design, School of Chemical Engineering, UNSW Sydney; NSW 2052, Australia; f Prince of Wales Hospital, School of Clinical Medicine, Randwick Clinical Campus, UNSW Sydney; NSW 2052, Australia; g The Kinghorn Cancer Centre, Garvan Institute of Medical Research; NSW 2010, Australia; h School of Clinical Medicine, St Vincent's Healthcare Campus, UNSW Sydney; NSW 2052, Australia; i Australian Pancreatic Cancer Genome Initiative (APGI), Garvan Institute of Medical Research; NSW 2010, Australia; j Cancer Diagnosis and Pathology Group, Kolling Institute of Medical Research, Royal North Shore Hospital; NSW 2065, Australia; k University of Sydney; Sydney, NSW 2006, Australia; l Mehmet Ali Aydinlar Acibadem University, Atakent University Hospital; Istanbul 34303, Turkey

**Keywords:** Pancreatic cancer, Chemosensitisation, Oxidative stress, DNA repair

## Abstract

Pancreatic ductal adenocarcinoma (PDAC) has a poor 5-year survival rate of just 13 %. Conventional therapies fail due to acquired chemoresistance. We previously identified MutY-Homolog (MYH), a protein that repairs oxidative DNA damage, as a therapeutic target that induces apoptosis in PDAC cells. However, we did not understand the mechanism driving these anti-PDAC effects, nor did we have a means to therapeutically inhibit MYH. In this study, we demonstrated that MYH inhibition induces DNA damage and checkpoint activation in PDAC cells. Using a clinically-relevant PDAC mouse model, we showed that therapeutic MYH-siRNA delivery using Star 3 nanoparticles increased intratumoural PDAC cell death, but did not inhibit tumour growth. Finally, we showed that MYH knockdown in PDAC cells sensitised them to the anti-proliferative and anti-clonogenic effects of oxaliplatin and olaparib. Our findings identify a potential novel therapeutic approach for PDAC that induces a therapeutically exploitable DNA repair vulnerability.

## Introduction

**Fig. 1:**
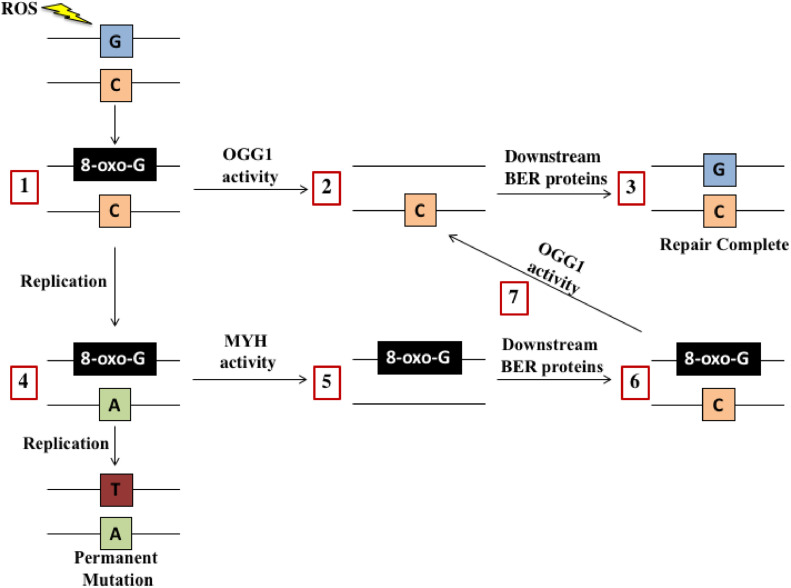
**Base excision repair (BER) of 8-oxoguanine in DNA.** The repair of 8-oxoguanine in DNA is primarily coordinated by the BER pathway glycosylases OGG1 and MYH. OGG1 detects and removes 8-oxoguanine in 8-oxoguanine:C mispairs **(1)**. This forms a non-coding abasic site **(2)** that is cleaved by the enzyme apurinic/apyrimidinic endonuclease 1 (APE1). This cleavage generates a single-strand DNA break that is then protected from further damage by scaffolding proteins. A DNA polymerase is then recruited to restore the correct DNA base. BER is completed by a ligase that repairs the single-strand DNA break **(3).** However, if the 8-oxoguanine:C mispair is not repaired before DNA replication, DNA polymerases can incorrectly insert an adenine (A) base opposite 8-oxoguanine to form and 8-oxoguanine:A mispair **(4)**. If left unrepaired, this can cause a permanent mutation. However, MYH recognises and excises this adenine (A) base from the DNA **(5)**. MYH then directs downstream BER proteins to restore an 8-oxoguanine:C mispair **(6)**. As described, 8-oxoguanine:C mispairs are acted upon by OGG1 **(7)**before BER proteins complete the DNA repair **(3). Abbreviations:** apurinic/apyrimidinic endonuclease 1 (APE1); base excision repair (BER); reactive oxygen species (ROS); MutY-Homolog (MYH); 8-oxoguanineuanine glycosylase (OGG1); 8-oxoguanineuanine (8-oxoguanine); adenine (A); thymine (T); guanine (G); cytosine (C).

Pancreatic ductal adenocarcinoma (PDAC) has a poor 5-year survival rate of just 13 %.^1^ Surgical resection provides the only potential cure, but the majority of patients present with advanced disease due to PDAC's non-specific symptoms and metastatic spread.^2^ As a result, most patients rely on chemotherapy.^2^ Despite this, our best chemotherapeutics only extend life by 6-11 months due to PDAC's significant chemoresistance ^3^. Therefore, we urgently need novel treatment strategies for PDAC. We previously identified MutY-Homolog (MYH) as a potential therapeutic target in PDAC.^4^ MYH is a member of the Base Excision Repair (BER) pathway that plays an instrumental role in repairing oxidised guanines (8-oxoguanine) in genomic DNA ^5^ (Fig. 1). This type of DNA damage is induced by oxidative stress (reactive oxygen species; ROS), which can be elevated in PDAC due to the Warburg effect that decreases intracellular anti-oxidants, cancer cell signaling, and chemotherapeutics that can induce oxidative stress in addition to their primary function .^6^ If left unrepaired, genomic 8-oxoguanine can be mispaired with adenine (A) during DNA replication, forming an 8-oxoguanine:A mispair that can become a permanent G:C to T:A mutation ^7^^,^^8^ (Fig. 1). The accumulation of these mutations can impair the expression of key survival genes, leading to cell death .^9^ Processing of 8-oxoguanines in close proximity and on opposite DNA strands by patch-excision DNA repair pathways (such as mismatch repair [MMR]), can also cause double-strand DNA breaks (DSBs) that can be fatal for cancer cells .^10^ Proteins that prevent and/or repair oxidative DNA damage are thus potential survival factors for PDAC cells. Our prior work showed that knockdown of MYH using siRNA reduced PDAC cell proliferation, increased apoptosis, and reduced metastatic potential *in vitro .*^4^ MYH knockdown via intratumoural siRNA delivery, also significantly reduced tumour growth in a subcutaneous mouse model of PDAC .^4^ However, we still did not fully understand the mechanism driving these anti-PDAC effects and whether they persisted in a more physiologically relevant setting and in the presence of cancer-associated fibroblasts (CAFs). CAFs are key perpetrators of PDAC fibrosis (physical barrier to drug delivery) and directly support PDAC cells .^11^^,^^12^

PDAC is also highly resistant to monotherapies, due to acquired chemoresistance. Herein we assessed functionally complementary therapeutic combinations with MYH knockdown: oxaliplatin, olaparib and MutT-homolog 1 (MTH1) inhibition. Oxaliplatin is a platinum-based chemotherapy that increases the production of reactive oxygen species (ROS) by disrupting the electron transport chain .^13^^,^^14^ Oxaliplatin is a constituent of FOLFIRINOX, a first-line combination treatment (folinic acid, 5-fluorouracil, irinotecan and oxaliplatin) used to treat PDAC .^15^ Oxaliplatin's application in the clinic and its ability to induce oxidative stress in addition to DNA adduct generation, made this a biologically and clinically relevant drug to test in combination with MYH inhibition. Olaparib inhibits poly-ADP ribose polymerase (PARP), a base excision DNA repair protein that binds and protects single-strand DNA breaks (SSBs) .^16^ PARP inhibition increases the frequency of SSBs that can become double strand DNA breaks (DSBs) .^17^^,^^18^ Inhibition of PARP on the right molecular background, for example BRCA1/2 mutated cells that are deficient in DSB repair, can lead to cell death .^17^^,^^18^ MYH knockdown had the potential to synergise with olaparib, as its absence could have led to increased incidence of SSBs generated by OGG1 and APE1 activity, in its absence .^19^ MTH1 is a protein that detects and degrades 8-oxoguanine in the nucleotide pool before they are mis-incorporated into genomic DNA .^20^ MTH1 is overexpressed in PDAC and is associated with poor overall survival .^21^ Evidence suggests that a dependence on MTH1 emerges in conditions of elevated intracellular oxidative stress, allowing cancer cells to exploit pro-tumourigenic ROS-signaling while avoiding potentially fatal ROS-induced DNA damage .^10^^,^^22^ As most non-tumour cells do not experience the same chronically increased oxidative stress as tumour cells, MTH1 represents a promising target to kill cancer cells while avoiding toxicity to normal cells .^23^^,^^24^ Studies have investigated MTH1 inhibition as a potential therapeutic strategy in PDAC using a range of chemical inhibitors .^25^^,^^26^ The complementary roles of MYH and MTH1 in preventing and repairing 8-oxoguanine in DNA provided a strong rationale to combine inhibition of these targets .^5^^,^^20^^,^^27^ This concept was investigated in T-cell acute lymphoblastic leukaemia cells and showed that dual knockdown of MTH1 and MYH induced apoptosis to a greater extent than knockdown of either target alone .^28^

In this study, we showed that MYH knockdown induced DNA damage in PDAC cells, but conversely reduced DNA damage in CAFs by inducing senescence. Using an orthotopic co-injection model of PDAC cells and CAFs in mice, we showed that systemic delivery of MYH-siRNA using Star 3 nanoparticles ^29^ increased intratumoural cell death, despite not reducing overall tumour growth. We subsequently demonstrated that MYH knockdown sensitised PDAC cells to the anti-clonogenic and anti-proliferative effects of oxaliplatin and olaparib but showed no additive effect with MTH1 knockdown. Our findings open the avenue for future development of these combination therapies for PDAC.

## Materials and Methods

### Cell culture

Human PDAC cells (MiaPaCa-2, Panc1, AsPC1, HPAFII) sourced from American Tissue Culture Collection were cultured as described .^30^^,^^31^ Cell line purity was confirmed using short tandem-repeat profiling (CellBank Australia). Genomically characterised patient-derived PDAC cells (TKCC5, TKCC10) were provided by Marina Pajic (Garvan Institute of Medical Research) and cultured as described .^32^ Patient-derived CAFs were isolated from surgically resected fibrotic tissue obtained from PDAC patients and cultured as described .^30^^,^^31^ CAF purity was confirmed by positive staining for glial fibrillary acidic protein (GFAP) and α-smooth muscle actin (αSMA) and negative staining for cytokeratin .^30^^,^^31^ Patient-derived CAFs were used within 12 passages of isolation and were approved by institutional human ethics committees (approvals: HC180973, UNSW Sydney, Australia; 5510/12, Technical University of Munich, Germany). All patients provided written informed consent. Cells were confirmed to be mycoplasma-negative (monthly). Commercial PDAC cells (MiaPaCa-2, Panc-1, HPAFII, AsPC1) were passaged using 0.25 % Trypsin/0.53 mM EDTA. TKCC cells and CAFs were passaged using 0.05 % Trypsin/0.53 mM EDTA.

### Western blot

Adhered cells were scraped into 1x Cell Lysis Buffer (Cell Signaling Technology, Danvers, Massachusetts, USA) containing 1 mM phenylmethanesulfonylfluoride Protein concentration was determined using Pierce BCA Kit (ThermoFisher Scientific, Cat. 23225) using manufacturer's instructions. 15μg of protein was electrophoresed on an SDS polyacrylamide gel (4 % stacking gel, 12 % resolving gel; 100 V) and then transferred to a 0.45μm nitrocellulose membrane (100 V, 1 h) as previously described .^33^ The membrane was blocked with 5 % skim milk powder in PBS/0.1 % Tween-20 (PBST) for 1 h at room temperature and probed with antibodies against the following targets:

i) MYH (1:1000) mouse monoclonal antibody (Abnova, Cat. H00004595-M01).
ii) Phosphorylated Chk1 (1:1000) rabbit monoclonal antibody (Cell Signaling, Cat.2348).
iii) Phosphorylated Chk2 (1:500) rabbit polyclonal antibody (Cell Signaling, Cat2661).
iv) MTH1 (1:1000) monoclonal mouse antibody (Santa Cruz Biotechnology, Cat. sc-271082).
v) OGG1 (1:1000) rabbit polyclonal antibody (Novus Biologicals, Cat. NB100-106).
vi) MSH2 (1:1000) rabbit polyclonal antibody (Abcam, Cat. 70270).
vii) GAPDH (1:50000) monoclonal mouse antibody (Abcam, Cat. Ab8245).
viii) α-tubulin (1:1000) monoclonal mouse antibody (Sigma-Aldrich, Cat. T9026).

Primary antibodies were detected with horseradish peroxidase (HRP)-linked polyclonal goat anti-mouse IgG (DAKO, Cat. PO447) or HRP-linked polyclonal goat anti-rabbit IgG (DAKO, Cat. PO448) diluted in 5 % skim milk/PBST, for 1 h at room temperature. Protein bands were visualised using Amersham ECL Western Blotting Detection Reagent (GE Healthcare Life Sciences, Cat. 2232) on an ImageQuant LAS4000 luminometer (GE Healthcare Life Sciences, Cat. 28955810) and quantified using ImageStudioLite software (LI-COR Biosciences) and standardised to housekeepers (GAPDH or α-tubulin).

### Single and dual silencing of MYH and MTH1 expression with short-interfering RNA

PDAC cells were transfected with lipofectamine 2000 (ThermoFisher) and 100 nM siRNA (or 50 nM of each siRNA in dual knockdown experiments) 24 h after seeding (10^5^ cells/well) in 6-well plates as described .^33^ OnTarget Plus siRNA (pool of four siRNA sequences) against MYH (Dharmacon, Cat. l-008260-00L-020099-00) and MTH1 (Dharmacon, Cat. AM16708) were used. Non-silencing siRNA (Dharmacon, Cat. d-001810-10-20) was used as a control. Knockdown was assessed 96 h post-transfection.

### Preparation of drugs

Oxaliplatin (Medchemexpress, HY-17371) and Olaparib (Medchemexpress, HY-10162) were dissolved in DMSO and stored at −80°C. T*ert*-Butyl hydroperoxide (tBHP; Sigma-Aldrich, Cat. 458139) was stored at 4°C. All drugs were diluted directly into complete culture medium for working concentrations.

### Quantification of cell proliferation and viability

MiaPaCa-2/AsPC1 PDAC cells were treated with or without tBHP (doses which reduced proliferation by approx. 50 %) for 48 h post-transfection. We measured AsPC1 and MiaPaCa-2 proliferation using an xCELLigence® Real-Time Cell Analysis (RTCA) instrument (ACEA Biosciences) or on the IncuCYTE ® S3 (Sartorius) platform.

### Determining the effect of single and dual MYH/MTH1 knockdown on pancreatic cancer cell clonogenicity and chemosensitivity

PDAC cell sensitivity to oxaliplatin (Focus Bioscience, Cat. HY-17371) and olaparib (MedChem Express, Cat. HY-10162) was assessed by clonogenic assay as previously described .^33^ MiaPaCa-2 PDAC cells were seeded in 6 well plates (10^5^ cells/well) and transfected with non-silencing siRNA, MYH-siRNA and/or MTH1-siRNA. 24 h post-transfection, MiaPaCa-2 cells were lifted and re-seeded into 6-well plates at 300 cells/well. 48 h post-transfection, cells were incubated with drug for 72 h. Colonies were allowed to form for a further 7-10 days (defined as >50 cells) in normal culture medium, and then fixed/stained with 0.5 % crystal violet (Sigma-Aldrich, Cat. 548-62-9) in 50 % methanol. Colonies were counted using an ImageQuant LAS4000 digital imager (GE Healthcare) and analysed with ImageQuant software.

### Apoptosis assay

PDAC cells were seeded and transfected as per section 2.3. 48 h post-transfection, cells were treated with 40μM tBHP or 7μM oxaliplatin for a further 48 h. Cells were stained with AnnexinV-FITC and DAPI as described .^33^ Total apoptosis was then quantified on a BD LSRFortessa X-20 as described .^33^

### Immunofluorescence measurement of γH2AX foci (DNA damage)

PDAC cells were seeded into 8-well chamber slides (Ibidi, Cat. 80806) 48 h post-transfection (MiaPaCa-2: 6000 cells/well; AsPC1: 10,000 cells/well). Cells were fixed in 4 % paraformaldehyde in 1x PBS (10 min at room temperature) 24 h later. Fixed cells were permeabilised with 0.5 % Triton-X in 1x PBS (10 min at room temperature) then blocked with 10 % goat serum in 1x PBS (30 min at room temperature). Cells were then incubated in Rabbit anti-γH2AX (Cell Signaling, Cat. 9718; 1:100) in 1 % Goat Serum/1x PBS for 1 h at room temperature. Washes between blocking and stains were performed with 0.1 % Tween/1x PBS for 3 × 5 min. Cells were then incubated in Goat anti-rabbit-AlexaFlour488 (Molecular Probes - A11008; 1:500) in 1 % goat serum/1x PBS in dark for 40 min at room temperature. Cells were mounted with ProLong Gold antifade reagent with DAPI (TheroFisher Scientific, Cat. P36941), then z-stacks (0.7 μm) of ≥4 representative regions per well (40x objective) that encompassed full nuclei were acquired on a Zeiss LSM 800 inverted confocal microscope**.** Maximum intensity projections were compiled in ZEISS Zen Black Software then γH2AX foci quantified per nucleus using particle analysis in ImageJ software.

### Oxidative stress (CellROX) assay

PDAC cells were seeded and transfected as per section 2.3. 48 h post-transfection, cells were treated with 40-80μM tBHP for a further 24 h. Cells were then stained with CellROX Green reagent (ThermoFisher Scientific, Cat. C10444) as per manufacturer's instructions. Intracellular fluorescence was then quantified on a BD LSRFortessa X-20.

### Cell senescence assay

CAFs were seeded 24 h post-transfection with siRNA, at 1500 cells/well in a 96-well plate. Cell senescence was assessed 96 h post-transfection using a Senescence β-galactosidase Cell Staining Kit (Cell Signaling Technologies, Cat. 9860) according to manufacturer's instructions. Senescent cells (blue) were quantified in ImageJ software, from 5 representative bright field photos (20x objective) of each well.

### Survival correlation analysis of MYH protein in human PDAC tissue microarrays

Formalin-fixed, paraffin-embedded human PDAC tissue microarrays (TMAs) were obtained through the APGI (International Cancer Genome Consortium Cohort; patient demographics in **Supplementary** Table 1). All patients provided written informed consent. TMA rehydration, blocking and immunohistochemistry for MYH was performed as described ^33^ using anti-MYH mouse monoclonal antibody (1:50 Overnight, 4°C; Abnova, Cat. H00004595-M01) and anti-mouse IgG secondary antibody (DAKO, Cat. E0433). The intensity of MYH staining in the tumour and stromal compartments was scored using four-point scales (0-3) by two independent scorers, based on intensity in ≥75 % of each compartment (normal acinar/ductal cells excluded). Note that tumour and stromal scoring scales were independent of each other. A consensus score was determined for each core, then for each set of 3 cores per patient, the highest scores in each compartment were used for survival analyses. Scores of 0-1 = MYH^low^; Scores of 2-3 = MYH^high^. Overall survival correlations were then performed using a Kaplan Meier Survival Curve. Patients still alive or deceased due to other causes were censored. Non-PDAC tumours, as well as patients without tumour in all 3 cores were excluded.

### Orthotopic PDAC mouse model

All animal experiments were approved by UNSW Animal Ethics committee (ACEC 18/54B). Orthotopic PDAC mouse models were established and performed as described .^33^ Briefly, luciferase-expressing MiaPaCa2 cells and PDAC patient-derived CAFs (10^6^ cells each) were implanted into the tail of the pancreas of mice. Tumours were grown for 4 weeks, then mice were randomized based on luminescence as described .^33^ Star 3+control siRNA (antisense: 5′-GAACUUCAGGGUCAGCUUGCCG) or MYH-siRNA single sequence (antisense: 5′-CGGAAGAGGUGGUAUUGCA) was then intravenously injected at 4 mg/kg twice weekly for 4 weeks. Primary tumour volume at endpoint was measured using micro-calipers and the formula volume = (length x width x height)/2. Tumours were subsequently fixed in 4 % paraformaldehyde and paraffin-embedded or snap-frozen in OCT, then sectioned as described .^33^

### Immunohistochemistry and immunofluorescence analysis of orthotopic PDAC tumour sections

***Immunohistochemistry: TUNEL (TdT-mediated dUTP-X nick end labeling; apoptosis/cell death marker)****:* Antigen retrieval and tissue blocking was performed on paraformaldehyde-fixed and paraffin-embedded orthotopic PDAC tumour sections as described .^33^ TUNEL staining was performed on tumour sections using In Situ Cell Death Detection Kit, Fluorescein (Sigma-Aldrich, Cat. 11684795910) according to manufacturer's instructions. TUNEL signal was visualised with Fast Red substrate (Thermo Fisher Scientific, Cat. TA-060-A) or by fluorescein fluorescence. Slides were scanned on a Vectra Polaris (PerkinElmer) slide scanner using a 40x/0.75 NA objective. Staining was quantified from whole tumour sections using QuPath software. ***Immunofluorescence:*** Immunofluorescence for αSMA was performed on OCT-embedded sections. Frozen tumour sections were brought to room temperature (10 min) then fixed for 10 min in 4 % paraformaldehyde (room temperature). Immunofluorescence staining was then performed for αSMA as described ^33^ using αSMA mouse antibody (Sigma-Aldrich, A5228; 1:1000 in 1 % goat serum/1xPBS, 1 h at room temperature) and goat anti-mouse-AF647 (1:500 in 1 % goat serum/1xPBS, 1 h at room temperature). Stained sections were mounted with ProLong Gold antifade reagent with DAPI (ThermoFisher Scientific, Cat. P36941) then scanned as above and staining quantified from ≥4 representative regions (average coverage = 10 %) using QuPath software.

### Statistical analysis

All statistical analysis was performed using the GraphPad Prism 9 software. Data was presented as mean + *S*.E.M (standard error of the mean). Independent replicates for all experiments were *n* ≥ 3. A Student t-test (2 groups) or one-way ANOVA with a parametric Bonferroni multiple comparison correction (≥3 groups) was used to determine statistical significance. Patient survival correlations were performed by log-rank test. *p* values < 0.05 were considered statistically significant.

## Results

### MYH Knockdown in PDAC cells induces DNA damage and cell cycle checkpoint activation

**Fig. 2:**
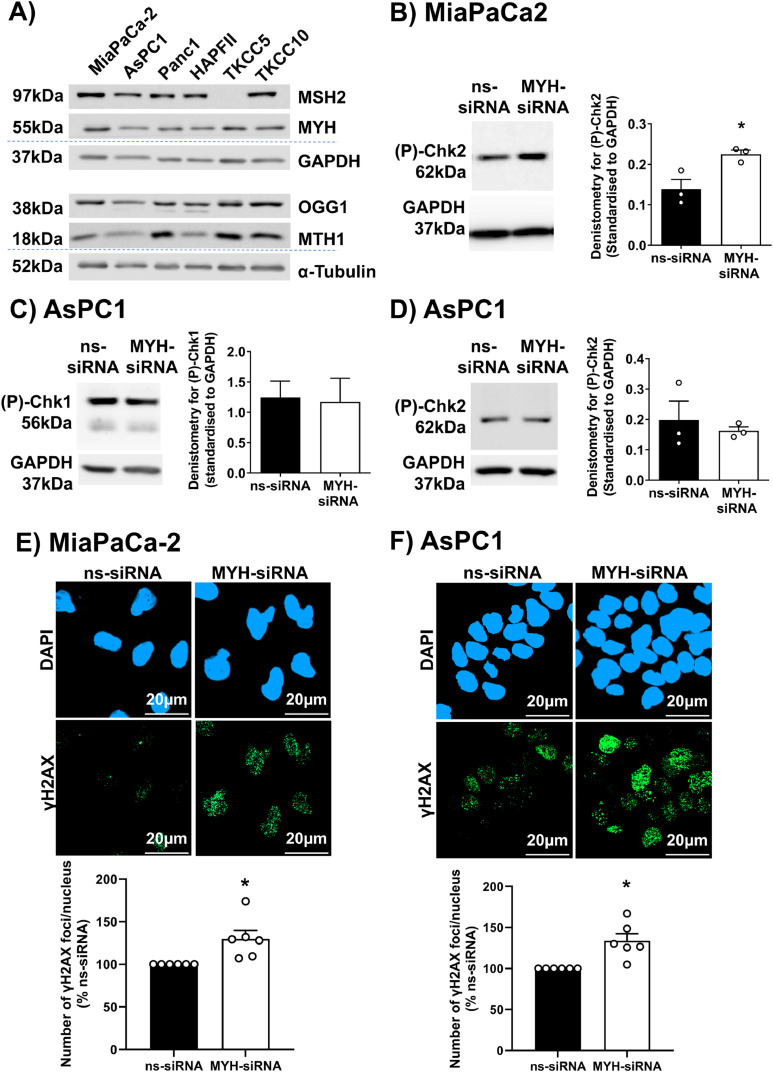
**MYH Knockdown in PDAC cells induces DNA damage. A)** Western blot comparing proteins involved in protection from oxidative DNA damage (MTH1: nucleotide pool sanitizer; MSH2: mismatch repair; OGG1, MYH: base excision repair glycosylase) in multiple PDAC cells. Alpha-tubulin was used as housekeeper for OGG1 and MTH1. GAPDH was housekeeper for MYH and MSH2. **B-D)** Western blots for phosphorylated Chk1 (P-Chk1) and Chk2 (P-Chk2) proteins in **(B)** MiaPaCa2 cells and **(C-D)** AsPC1 cells transfected with non silencing-siRNA (ns-siRNA) or MYH-siRNA. GAPDH was used as a housekeeper. Note that P-Chk1 was not detectable in MiaPaCa2 cells. Graphs show densitometry for P-Chk1 and P-Chk2 bands. **E-F)** Representative photos of immunofluorescence for gammaH2AX (DNA damage) and DAPI (nuclei) in **(E)** MiaPaCa2 cells and **(F)** AsPC1 cells transfected with ns-siRNA or MYH-siRNA (γH2AX foci per nucleus as a percent of ns-siRNA). Symbols in graphs indicate independent replicate experiments. Bars and lines = mean+s.e.m. Asterisks indicate significance (*p ≤ 0.05, student t-test).

We assessed the expression of OGG1, MYH (BER glycosylase), MSH2 (MMR initiator) and MTH1 (nucleotide pool sanitiser), proteins related to protection from genomic 8-oxoguanine, across multiple PDAC cells (Fig. 2A). We found that DNA repair proteins are present at variable levels across PDAC cells, with MiaPaCa2, Panc1 and TKCC10 having the highest levels across all detected proteins (Fig. 2A). As expected, the MMR protein MSH2 was not expressed in the MMR deficient TKCC5 PDAC cells (Fig. 2A**)**.^32^ AsPC1 and HPAFII had the lowest levels of MYH and MTH1, compared to other tested PDAC cells (Fig. 2A). Our PDAC cells could largely be grouped into those with high expression of 8-oxoguanine DNA repair proteins (MiaPaCa2, PANC1, TKCC10) and those with deficiencies/low expression in at least one of these proteins (AsPC1, HPAFII, TKCC5). MiaPaCa-2 and AsPC1 were used as representatives of these groups for all subsequent analyses. Knockdown was confirmed by Western blot in both cell lines (**Supplementary** Figure 1). Interestingly, while MYH knockdown did not affect MTH1 protein levels, in MiaPaCa2, MTH1 knockdown halved MYH protein level, suggesting a potential feedback loop between MTH1 and MYH in this cell line (**Supplementary** Figure 1). In previous work, we showed that MYH knockdown significantly reduced PDAC cell proliferation in both MiaPaCa2 and AsPC1 PDAC cells by inducing apoptosis and altering cell cycle progression .^4^ We investigated whether DNA damage was the driving mechanism behind these effects by assessing activation (phosphorylation) of DNA damage-related checkpoint proteins Chk1 and Chk2, as well as direct DNA damage (γH2AX foci). In MiaPaCa2 cells, MYH knockdown significantly increased Phospho-Chk2 relative to controls (Figure 2B; note that phospho-Chk1 was undetectable). In contrast, MYH knockdown in AsPC1 cells had no effect on Phospho-Chk1 or Phospho-Chk2 levels, relative to controls (Fig. 2C-D). Quantification of γH2AX foci (marker of DNA double strand breaks; ^34^) showed that MYH knockdown significantly increased DNA damage in both MiaPaCa2 and AsPC1 cells (Fig. 2E-F). This effect was not due to MYH knockdown reducing antioxidant capacity (**Supplementary** Figure 2A), reinforcing that observed effects were due to interference with its DNA repair role.

### Therapeutic MYH knockdown in orthotopic PDAC tumours increased intratumoural cell death and immune infiltrate

**Fig. 3:**
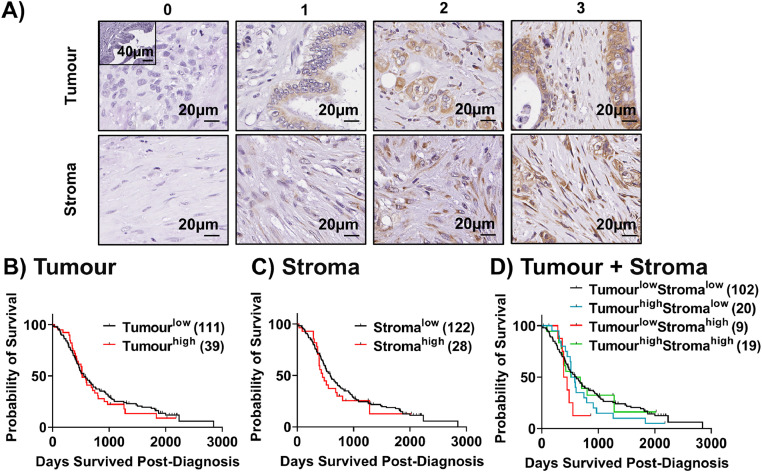
**MYH is a potential therapeutic target in PDAC CAFs, but its expression in the tumour and stroma does not predict patient survival. A)** Reference images for scoring of MYH expression (tumour and stroma scored on separate scales) in the Australian Pancreatic Cancer Genome Initiative International Cancer Genome Consortium PDAC cohort. **B-D)** Survival curves for patients expressing high (scores 2-3) versus low (scores 0-1) MYH expression in the (**B)** tumour compartment, **(C)** stroma compartment or **(D)** both. Numbers in brackets indicate the number of patients per group.

**Fig. 4:**
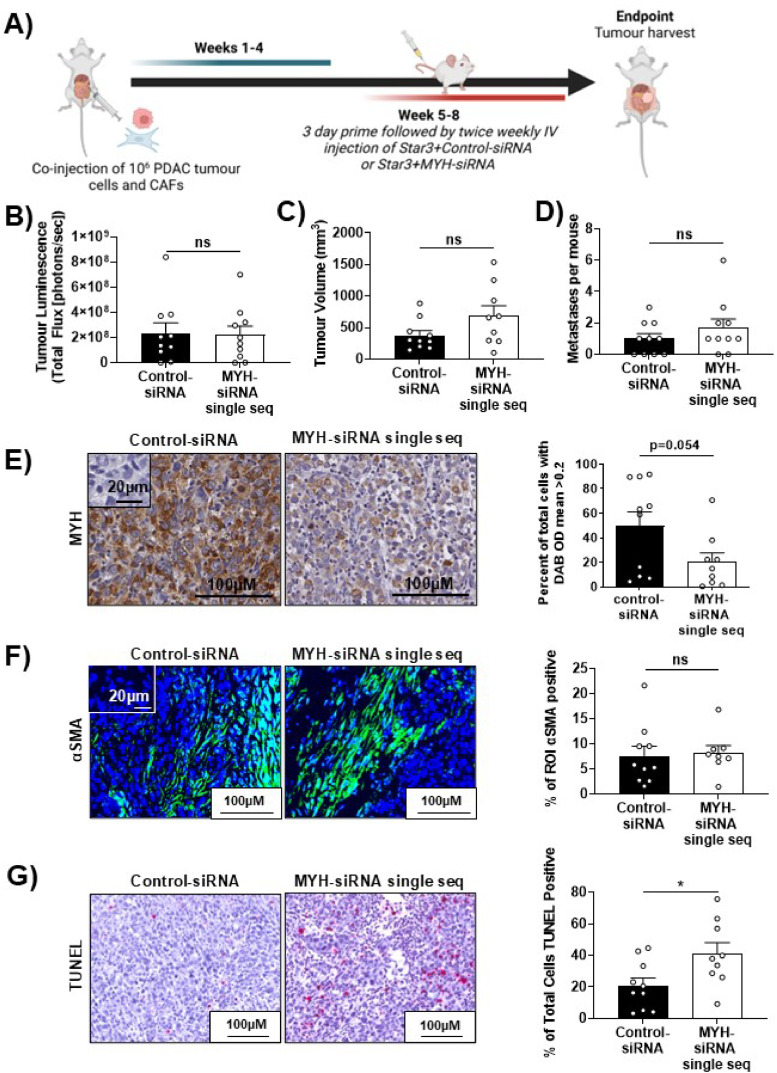
**Therapeutic MYH knockdown in PDAC tumours increased intratumoural cell death.** All orthotopic tumours were co-injections of MiaPaCa-2 PDAC cells and human primary patient-derived CAFs. **A)** Model overview. **B)** Tumour luminescence in standardised groups at start of treatment. **C)** Tumour volume at therapeutic model endpoint, as assessed by calliper measurement (*n* = 8-9). **D)** Quantification of metastatic sites per mouse at model endpoint based on ex vivo luminescence (*n* = 8-9). **E-G)** Representative images and quantification of **(E)** MYH (inset = isotype control), **(F)** αSMA (CAFs, green; blue = nuclear stain; inset = isotype control; quantification of the average % aSMA positive area per region of interest [ROI], per mouse), **(G)** TUNEL (cell death, red). Lines in all graphs = mean ± s.e.m. Asterisks in all graphs indicate significance (ns: not significant, *p ≤ 0.05; student t-test). Panel A created with BioRender.com.

We assessed the prognostic significance of MYH protein levels in human pancreatic tumour specimens (APGI tissue microarrays). We found that in both tumour and stroma, MYH expression was low in the majority (74 % for tumour; 81 % for stroma) of PDAC patients in this cohort, and that expression did not correlate with survival (Fig. 3B-D). We investigated the impact of MYH knockdown on PDAC tumours in vivo, using an orthotopic PDAC mouse model (luciferase expressing MiaPaCa-2 and CAF co-injection). Mice were treated with Star 3 nanoparticles ,^29^ complexed to either control-siRNA or MYH-siRNA (Fig. 4A). Mice were randomised into two treatment groups based on tumour luminescence prior to treatment (Fig. 4B). We found that MYH knockdown had no effect on tumour size or metastatic spread (Fig. 4C-D). MYH knockdown was confirmed by immunohistochemistry (Fig. 4E). Treatment has no effect on CAF frequency relative to controls (αSMA; Fig. 4F). However, we did observe significantly increased intratumoural TUNEL staining (cell death) in the MYH-siRNA treated mice (Fig. 4G). Our findings ultimately highlighted the need for an effective combination therapy to enhance the impact of MYH knockdown against PDAC tumours.

### Individual MYH and MTH1 knockdown increased PDAC cell sensitivity to oxidative stress

**Fig. 5:**
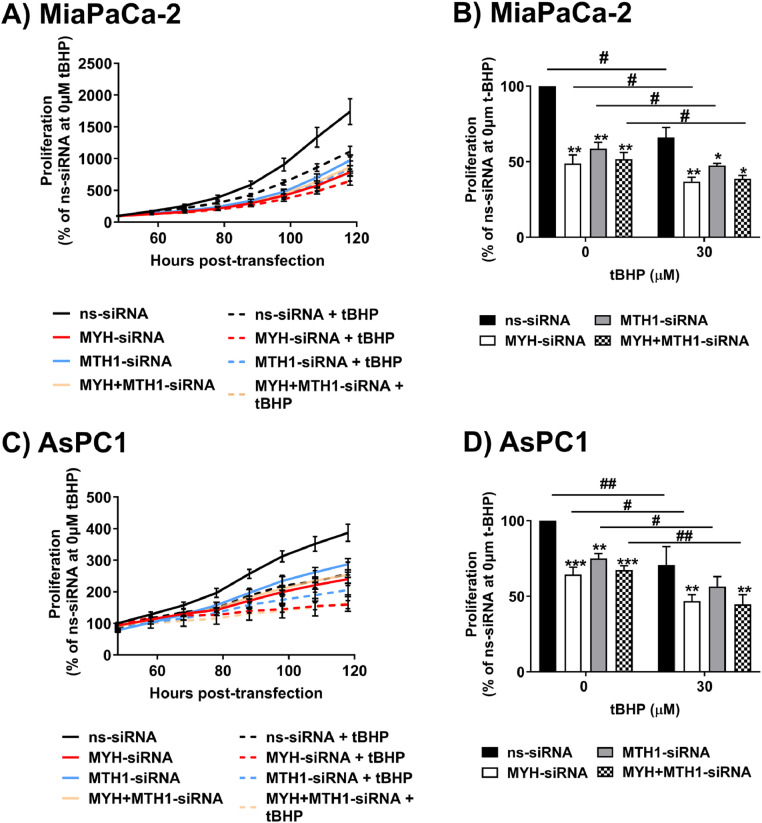
**MYH and MTH1 knockdown in PDAC cells increased their sensitivity to the anti-proliferative effects of tert‑butyl hydroperoxide (tBHP; oxidative stress). (A,C)** Line graphs show PDAC cell proliferation 48 h-120 h post-transfection with non silencing-siRNA (ns-siRNA) or MYH-siRNA, as measured on an xCelligence platform (expressed as % of ns-siRNA control). Treatment with tBHP occurred at 48 h and 72 h post-transfection. The quantification and statistical comparison of cell proliferation at 96 h (latest time point at which knockdown was confirmed) is shown in **(B,D)**. Bars and lines = mean+s.e.m (*n* = 4 independent replicates). Asterisks indicate significance relative to ns-siRNA at the same concentration of tBHP (*p ≤ 0.05, **p ≤ 0.01, ***p ≤ 0.001; one-way ANOVA). Hashes indicate significance relative to the 0μM tBHP treatment of the same siRNA (^#^*p* ≤ 0.05, ^##^*p* ≤ 0.01; one-way ANOVA).

We assessed if co-inhibition of MTH1 with MYH could further enhance PDAC cell sensitivity to oxidative stress, using an siRNA-based approach (single and dual knockdown) in the presence or absence of the oxidative stress inducer, tBHP. Dual knockdown was confirmed by Western blot in MiaPaCa-2 and AsPC1 cells (**Supplementary** Figure 1). In both cell lines, single knockdown of MTH1 or MYH significantly reduced cell proliferation and increased cell sensitivity to the anti-proliferative effects of tBHP, relative to controls (Fig. 5A-D; **Supplementary** Figure 2B). However, MTH1 and MYH dual knockdown did not reduce proliferation or increase sensitivity to oxidative stress any more than knockdown of either protein alone (Fig. 5A-D).

### MYH knockdown chemosensitised PDAC cells to oxaliplatin and olaparib

**Fig. 6:**
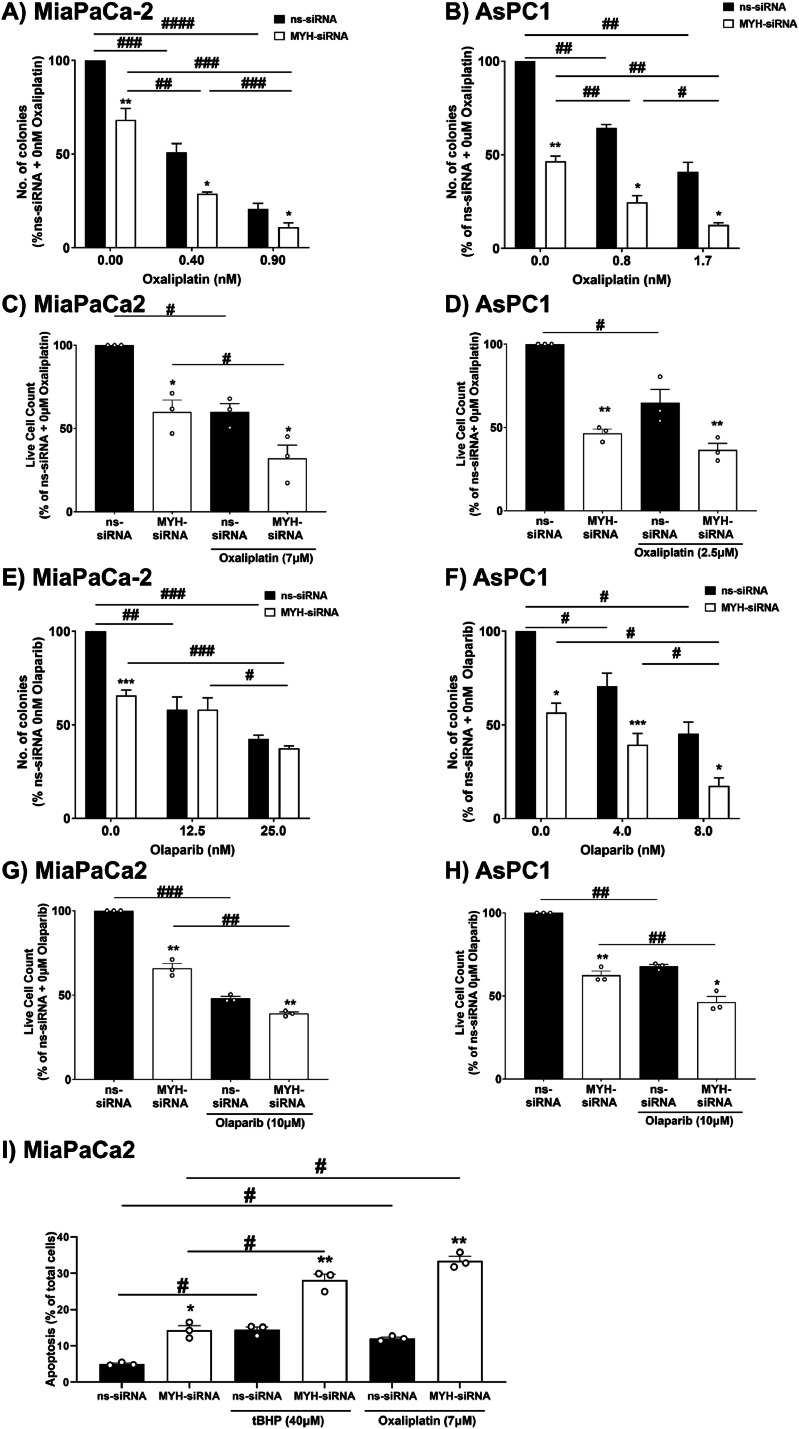
**MYH knockdown in PDAC cells increased sensitivity to oxaliplatin and olaparib.** Clonogenic assays were performed for **(A,E)** MiaPaCa2 and **(B,F)** AsPC1 post-transfection with non-silencing siRNA (ns-siRNA) or MYH-siRNA, and co-treatment with oxaliplatin (platinum drug and oxidative stress inducer) or olaparib (PARP inhibitor). Graphs show colony count (as a % of ns-siRNA 0μM drug control). **C-D, G-H)** As per **(A-B, E-F)** except a trypan blue exclusion assay was performed at 96 h post-transfection and 48 h post-drug treatment. **I)** As per **(A-B, E-F)** except apoptosis analysis (AnnexinV/DPAI staining and flowcytometry) was performed at 96 h post-transfection and 48 h post-drug treatment. Symbols indicate independent replicate experiments. Bars and lines = mean+s.e.m (**A-B, E-F)**, *n* = 3-5 independent replicates; **C-D, G-I***n* = 3 independent replicates). Asterisks indicate significance relative to ns-siRNA at the same concentration of drug or as indicated by lines (*p ≤ 0.05, **p ≤ 0.01, ***p ≤ 0.001; one-way ANOVA). Hashes indicate significance relative to the 0μM drug treatment of the same siRNA (^#^*p* ≤ 0.05, ^##^*p* ≤ 0.01, ^###^*p* ≤ 0.001, #### *p* ≤ 0.0001; one-way ANOVA).

We next assessed the effect of MYH knockdown on PDAC cell sensitivity to a chemotherapy agent known to induce oxidative stress (oxaliplatin) and a PARP inhibitor (olaparib), using clonogenic and trypan blue exclusion assays. MYH knockdown or oxaliplatin treatment alone reduced colony formation and viable cell count in both PDAC cell lines (Fig. 6A-D). Importantly, MYH knockdown increased MiaPaCa-2 and AsPC1 cell sensitivity to oxaliplatin in both assays (Fig. 6A-D). Likewise, MYH knockdown or olaparib treatment alone reduced colony formation and viable cell count in both MiaPaCa-2 and AsPC1 cell lines (Fig. 6E-H). Simlarly, MYH knockdown sensitised MiaPaCa-2 and AsPC1 cells to olaparib in trypan blue exclusion assays (Fig. 6G-H), but in contrast to oxaliplatin, in clonogenic assays MYH knockdown sensitised AsPC1 cells, but not MiaPaCa-2 cells, to the anti-clonogenic effects of olaparib (Fig. 6E-F). We further confirmed that MYH knockdown in PDAC cells significantly enhanced apoptosis induced by tBHP or oxaliplatin (Fig. 6I).

## Discussion

Approaches that exploit DNA repair deficiencies in cancer cells have had significant success in a variety of cancers, most notably PARP inhibitors in homologous recombination-deficient tumours .[35], [36], [37], [38] This study explored the potential to do the same in PDAC, but using an approach that targets the glycosylase MYH instead of an underlying mutation. Our work builds on our earlier findings that identified MYH as a potential therapeutic target in PDAC cells .^4^ In this study, we showed that MYH knockdown in PDAC cells reduces their DNA repair capability and sensitises them to DNA damaging chemotherapeutic agents. Our findings also importantly show that MYH knockdown as a monotherapy cannot reduce pancreatic tumour growth and metastasis *in vivo*, but promisingly increases intra-tumoural cell death. Taken together, these results suggest that a combination of MYH knockdown with appropriate DNA damaging agents may be a novel therapeutic avenue for PDAC, that warrants further *in vivo* investigation.

We tested the effects of MYH knockdown in an orthotopic PDAC mouse model that reproduces key features of clinical disease including fibrosis, metastatic spread and chemoresistance .^33^ Therapeutic MYH inhibition was achieved using Star 3 nanoparticles that our laboratory previously designed to deliver siRNA to pancreatic tumours after systemic administration .^29^^,^^33^ MYH knockdown did not reduce tumour growth/metastasis or intratumoural CAF frequency, but increased intratumoural cell death. The result may be reflective of a “double-edged sword” effect, where MYH knockdown may have benefited some subsets of PDAC cells that were enough to continue fueling tumour growth, especially with the support of CAFs. The contrast of these findings with our previous sub-cutaneous model, which showed MYH knockdown significantly reduced tumour growth, can be explained by the lack of CAFs and the sub-cutaneous location of our prior model, as well as the shorter time frame and intratumoural route for treatment .^4^ Our findings demonstrated that MYH knockdown required an appropriate combination therapy to enhance PDAC cell death and improve therapeutic efficacy against PDAC tumours.

To identify appropriate combination therapies, we needed a deeper understanding of how MYH inhibition affects pancreatic cancer cells. We showed that MYH knockdown in PDAC cells induces DNA double strand breaks but that the response to damage is cell line-dependent (that is, MiaPaCa-2: increased checkpoint activation; AsPC1: no checkpoint activation). The observed increase in DNA double strand breaks is consistent with MYH's role in repairing 8-oxoG:A mispairs. In the absence of MYH activity, the increased processing of closely situated 8-oxoguanine:A mispairs (e.g. by OGG1 and mismatch repair) increases the chances of generating DNA double strand breaks, which can induce cell cycle checkpoints, senescence, and apoptosis .^10^^,^^39^ In MiaPaCa-2 cells, unresolved checkpoint activation can eventually compromise cell survival. In the absence of checkpoint activation, AsPC1 cells likely succumb to unregulated accumulation of DNA damage and mutation induced by MYH knockdown. The results reinforce that the effects of MYH inhibition in pancreatic cancer cells are dependent on the DNA repair role of MYH and importantly, that the outcome regardless of checkpoint activation is reduced proliferation.

We initially assessed a combination of concurrent MYH and MTH1 knockdown. MTH1 inhibition increases the incorporation of 8-oxoguanine into DNA, making it possible that this approach would place greater demand on DNA repair pathways to avoid lethal levels of DNA damage. However, while individual knockdown of either MYH or MTH1 in PDAC cells significantly reduced their proliferation and increased their sensitivity to oxidative stress, dual inhibition was not more effective than MYH alone. This result might be a consequence of redundancies in MTH1 function, for example, the protein MTH2 could partly substitute for its activity .^40^ In contrast, OGG1 relies on MYH to complete repair .^41^ Interestingly, the anti-proliferative impact of MTH1 knockdown in PDAC cells that we observed was also less potent than previous studies, which utilised competitive inhibitors of the MTH1 binding pocket (TH287 and (S)-crizotinib) and demonstrated reductions of PDAC cell viability >70 % .^26^ This difference may be explained by residual MTH1 activity left by siRNA, as opposed to chemical inhibitors which can inhibit the entire pool of active protein .^26^ Alternatively, it may be explained by potential off-target effects of MTH1 inhibitors .^26^ For example, TH287 was shown to inhibit tubulin polymerisation – to induce apoptosis .^26^

We next turned our attention to functionally relevant therapeutics to combine with MYH inhibition: oxaliplatin and olaparib. Olaparib inhibits PARP, a component of the base excision DNA repair pathway that binds and protects DNA single strand breaks .^16^ PARP inhibition increases the frequency of SSBs that can become DSBs ,^17^^,^^18^ which is why it is effective in BRCA1/2 mutated cells that are deficient in DNA double strand break repair .^17^^,^^18^ This was demonstrated in a Phase III trial in patients with BRCA-mutated PDAC ,^42^ resulting in its approval as a maintenance therapy in this subset of PDAC patients. MYH plays an important role in coordinating OGG1, APE1 and downstream BER activities including PARP protection of single strand breaks at sites of 8-oxoguanine, to ensure the appropriate sequence of events to safely complete the repair .^19^ Olaparib thus had the potential to expose a greater frequency of OGG1/APE1-generated DNA single strand breaks to become lethal DNA double strand breaks in the absence of MYH. We indeed observed that MYH knockdown moderately sensitised MiaPaCa-2 and AsPC1 to the anti-proliferative effects of olaparib. However, in a clonogenic assay, which tests the ability of cancer cells to seed colonies from single cells, olaparib only enhanced the anti-clonogenic effects of MYH knockdown in AsPC1 PDAC cells. It may be that in a low-density setting, cellular changes in MiaPaCa-2 cells reduce their reliance on PARP or reduce DNA single strand breaks induced by MYH knockdown.

Our previous work had demonstrated that MYH knockdown in PDAC cells increased their chemosensitivity to gemcitabine and paclitaxel, which are first-line treatments for non-resectable PDAC in the clinic .^4^^,^^15^ However these chemotherapeutics are relatively low level inducers of oxidative stress .^43^ Oxaliplatin was selected as a stronger oxidative stress inducer ^13^^,^^14^ and as a component of FOLFIRINOX, a first-line chemotherapeutic regime used to treat PDAC patients .^15^ Promisingly, our clonogenic and proliferation results showed that MYH knockdown in both MiaPaCa-2 and AsPC1 PDAC cells sensitised them to oxaliplatin. We also confirmed that MYH knockdown sensitised PDAC cells to tBHP and oxaliplatin-induced apoptosis *in vitro*. Our results highlight the therapeutic potential of combining MYH inhibition with oxaliplatin or a similar oxidative stress inducer in PDAC.

Finally, we assessed the prognostic significance of MYH in human PDAC tissue from the APGI ICGC PDAC cohort. We showed that MYH expression was not predictive of survival in PDAC, and that in the majority of both tumour and stromal compartments, MYH protein levels were low. While MYH expression alone was not predictive of survival, it is important to acknowledge that MYH works alongside several other proteins involved in genomic surveillance and DNA repair .^44^ Future studies should assess the combined prognostic significance of MYH with other key oxidative DNA damage repair proteins.

The results from this study have deepened our understanding of how oxidative DNA damage repair is regulated in PDAC cells and provided critical information for future development of an MYH inhibition-based combination therapeutic approach. Our novel results have formed the foundation for further pre-clinical testing of MYH and using a novel nanomedicine-RNA interference-based approach in combination with clinically relevant chemotherapeutics (platinum-based drugs and PARP inhibitors) and has the potential for future clinical translation.

## Supplementary Materials

Figure S1: Confirmation of MYH and MTH1 knockdown in PDAC cells. Figure S2: CellROX and incucyte assays; Table S1: Australian Pancreatic Cancer Genome Initiative (APGI) International Cancer Genome Cohort (ICGC) patient characteristics for MYH survival analyses.

## Funding

This research was made possible by major funding from Cancer-Institute NSW CDF (G. Sharbeen, CDF181166), 10.13039/501100001184Cure Cancer Australia (G. Sharbeen, APP1122758), Tour de Cure Pioneering Research Grant (G. Sharbeen, P.A. Phillips, D. Goldstein UNSWR004; RSP-255-19/20), NHMRC Ideas Grant (P.A.Phillips, J.McCarroll, G.Sharbeen, APP2002707), Maridulu Budyari Gumal Sydney Partnership for Health, Education, Research and Enterprise [SPHERE] Cancer Clinical Academic Group Senior Research Fellowship (Funded by Cancer Institute NSW Translational Cancer Research Capacity Building Grant, 2021, G. Sharbeen), Pankind Innovation Grant (G. Sharbeen) Australian Government Research Training Program Scholarship & UNSW Sydney Scientia PhD Scholarship (J. Kokkinos), Cancer-Institute NSW Translational Program Grant (2020/TPG2100, D. Goldstein, P. Phillips, M Pajic).

## Institutional Review Board Statement

The study was conducted in accordance with the Declaration of Helsinki, and approved by the UNSW Sydney Ethics Committee (HC180973 approved 23/1/2019) and the Technical University of Munich, Germany (5510/12) for use of human specimens and patient-derived cell lines. The animal study protocol was approved by the UNSW Sydney Animal Ethics Committee (ACEC18/54B).

## Informed Consent Statement

Informed consent was obtained from all subjects involved in the study.

## CRediT authorship contribution statement

**James Ephraums:** Conceptualization, Investigation, Methodology, Writing – original draft, Writing – review & editing, Formal analysis. **Janet Youkhana:** Formal analysis, Investigation, Methodology, Writing – review & editing. **Aparna S. Raina:** Formal analysis, Investigation, Methodology, Writing – review & editing. **Grace Schulstad:** Formal analysis, Investigation, Methodology, Writing – review & editing. **Kento Croft:** Formal analysis, Investigation, Methodology, Writing – review & editing. **Amanda Mawson:** Formal analysis, Investigation, Methodology, Writing – review & editing. **John Kokkinos:** Formal analysis, Investigation, Methodology, Writing – review & editing. **Estrella Gonzales-Aloy:** Formal analysis, Investigation, Methodology, Writing – review & editing. **Rosa Mistica C. Ignacio:** Formal analysis, Investigation, Methodology, Writing – review & editing. **Joshua A. McCarroll:** Conceptualization, Funding acquisition, Investigation, Writing – review & editing. **Cyrille Boyer:** Investigation, Resources, Writing – review & editing. **David Goldstein:** Conceptualization, Funding acquisition, Investigation, Writing – review & editing. **Marina Pajic:** Investigation, Resources, Writing – review & editing. **Koroush S. Haghighi:** Data curation, Investigation, Resources, Validation, Writing – review & editing. **Amber Johns:** Data curation, Investigation, Resources, Validation, Writing – review & editing. **Anthony J. Gill:** Data curation, Investigation, Resources, Validation, Writing – review & editing. **Mert Erkan:** Data curation, Investigation, Resources, Validation, Writing – review & editing. **Australian Pancreatic Cancer Genome Initiative (APGI):** Data curation, Resources, Writing – review & editing. **Phoebe A. Phillips:** Conceptualization, Formal analysis, Funding acquisition, Investigation, Methodology, Project administration, Writing – review & editing. **George Sharbeen:** Conceptualization, Formal analysis, Funding acquisition, Investigation, Methodology, Project administration, Visualization, Writing – original draft, Writing – review & editing.

## Declaration of competing interest

The authors declare that they have no known competing financial interests or personal relationships that could have appeared to influence the work reported in this paper.

## Appendix Supplementary materials

Image, application 1
