# Supplementary material for: MYH knockdown in pancreatic cancer cells creates an exploitable DNA repair vulnerability
Source: Neoplasia. 2025 Feb 11;61:101138. doi: 10.1016/j.neo.2025.101138 (PMC11869960; doi:10.1016/j.neo.2025.101138)
Supplement: Supplementary file 1 [file mmc1.pdf]

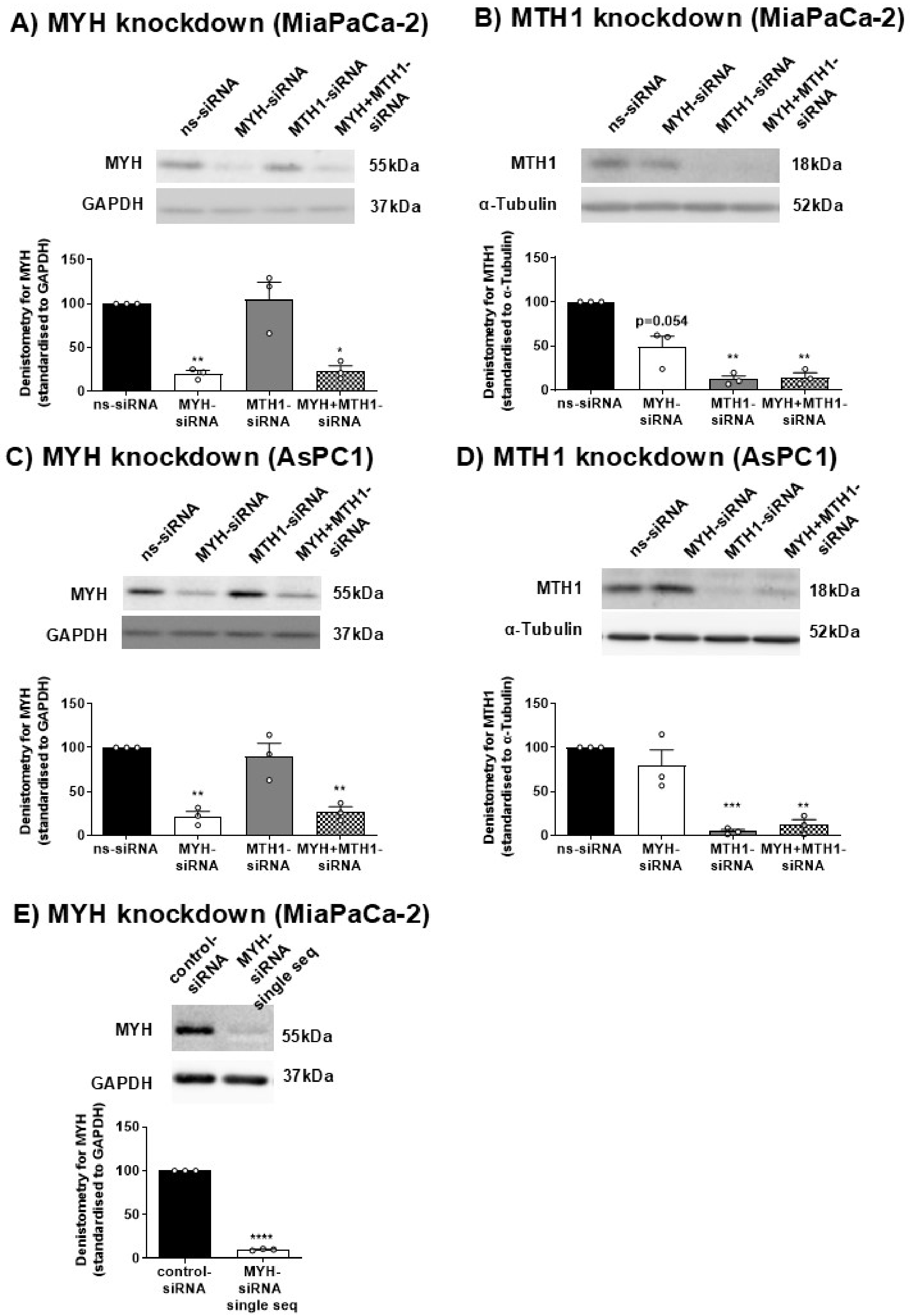

Supplementary Figure 1: Confirmation of MYH and MTH1 knockdown in PDAC cells and CAFs. *Representative Western blots and densitometry from three independent experiments are shown in each panel. GAPDH and α-Tubulin were used as housekeepers for standardisation. Lines in all graphs represent mean ± standard error of mean. Asterisks indicate significance (*p≤0.05, **p≤0.01, ***p≤0.001, ****p≤0.0001; one-way ANOVA [A-D] or student t-test [E]).*

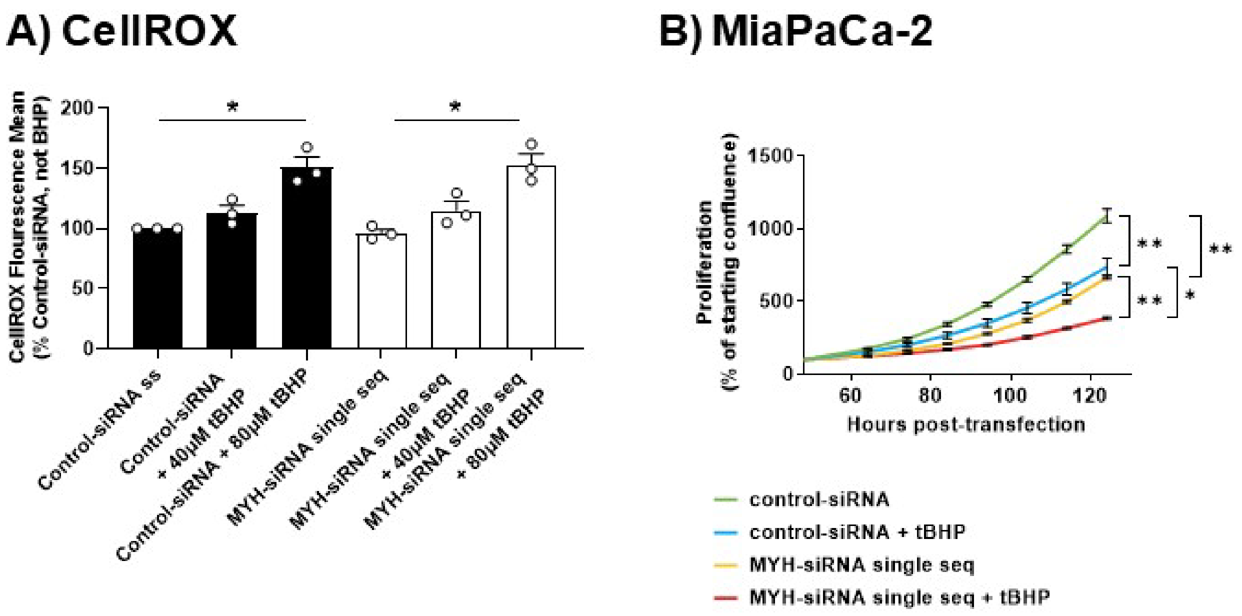

Supplementary Figure 2: CellROX and proliferation assays following MYH knockdown in PDAC cells. A) *Quantification of CellROX intracellular flourescence (oxidative stress) measured at 72 hours post-transfection (control single sequence siRNA [control-siRNA] or MYH single sequence siRNA [MYH-siRNA single seq]) and 24h post-treatment with tBHP. Symbols indicated independent experiments. Bars and lines indicate mean + s.e.m. *p≤0.05.* ***B)*** *Proliferation measured on an IncuCYTE ® S3 of MiaPaCa-2 post-transfection with control-siRNA or MYH-siRNA single seq, in the presence or absence of 40µM tBHP (added at start of imaging). Lines in all graphs represent mean ± standard error of mean for each time point. Asterisks indicate significance at endpoint (*p≤0.05, **p≤0.01; one-way ANOVA).*

**Age at diagnosis**	**Number of patients**
≥50	140
<50	11
**Gender**	
Male	83
Female	68
**Ethnicity**	
Asian	12
Asian, White/Caucasian	1
Black/African	1
Pacific Islander	1
White/Caucasian	136
**Smoker**	
Ever	81
Never	66
Not reported	4
**Alcohol consumption**	
Ever	90
Never	55
Not reported	6
**Margin Status**	
R0	98
R1	48
R2	3
RX	2
**Macroscopic tumour location**	
Ampulla	2
Body	11
Head	115
Head (Uncinate)	7
Tail	14
Not reported	2
**Overall Stage**	
IA	2
IB	1
IIA	32
IIB	109
III	0
IV	5
Not reported	2

**TNM Staging**	
T1	2
T2	5
T3	142
T4	0
TX	2
N0	35
N1	73
N1a	7
N1b	33
NX	3
M0	7
M1	5
MX	139
**Perineural invasion**	
Yes	126
No	22
Not reported	3
**Vascular invasion**	
Yes	95
No	50
Not reported	6
**Recurrence at liver**	
Yes	52
No	69
No recurrence	30

Supplementary Table 1: Australian Pancreatic Cancer Genome Initiative (APGI) International Cancer Genome Cohort (ICGC) patient characteristics for MYH survival analyses. *Human PDAC tissue microarrays were obtained through the APGI. Patient cohort characteristics are described above. TNM staging refers to tumour size (T), lymph node involvement (N), and metastasis (M).*
